# Progress of Research into Preformed Particle Gels for Profile Control and Water Shutoff Techniques

**DOI:** 10.3390/gels10060372

**Published:** 2024-05-28

**Authors:** Wei Ma, Yikun Li, Pingde Liu, Zhichang Liu, Tao Song

**Affiliations:** 1 Key Laboratory of Oilfield Chemistry of China National Petroleum Corporation, Research Institute of Petroleum Exploration and Development, PetroChina, Beijing 100083, China; mw17860271013@163.com (W.M.);; 2 College of Chemical Engineering and Environment, China University of Petroleum (Beijing), Beijing 102249, China; 3 National Elite Institute of Engineering, Beijing 100096, China

**Keywords:** preformed particle gels (PPGs), profile control and displacement, water shutoff, fracture

## Abstract

Gel treatment is an economical and efficient method of controlling excessive water production. The gelation of in situ gels is prone to being affected by the dilution of formation water, chromatographic during the transportation process, and thus controlling the gelation time and penetration depth is a challenging task. Therefore, a novel gel system termed preformed particle gels (PPGs) has been developed to overcome the drawbacks of in situ gels. PPGs are superabsorbent polymer gels which can swell but not dissolve in brines. Typically, PPGs are a granular gels formed based on the crosslinking of polyacrylamide, characterized by controllable particle size and strength. This work summarizes the application scenarios of PPGs and elucidates their plugging mechanisms. Additionally, several newly developed PPG systems such as high-temperature-resistant PPGs, re-crosslinkable PPGs, and delayed-swelling PPGs are also covered. This research indicates that PPGs can selectively block the formation of fractures or high-permeability channels. The performance of the novel modified PPGs was superior to in situ gels in harsh environments. Lastly, we outlined recommended improvements for the novel PPGs and suggested future research directions.

## 1. Introduction

As oil field development progresses, the production rates of oil wells gradually decrease. Existing techniques to enhance oil production rate mainly include fracturing [1], acidizing [2], profile control, and water shutoff. Excessive water production can lead to reduced oil production or even well abandonment, primarily due to reservoir heterogeneity and especially due to the presence of high-permeability fractures. Profile control and water shutoff is a crucial technology in oil field development for enhancing sweep efficiency and reducing water invasion in formations. Gel treatment is an economical and efficient method for controlling excessive water production and has been widely applied in the field. Several polymer gel systems have been developed, including in situ gels and PPGs. In situ gel treatment involves the simultaneous or sequential injection of polymer and crosslinker solutions, termed gelants, into the formation. Gelants can change to gels under the stimuli of temperature or salinity to block high-permeability channels. This method has several inherent drawbacks; to be more precise, the gelation time, gel quality, and injection depth are difficult to precisely control. Moreover, it may potentially damage the reservoir, resulting in the simultaneous plugging of oil and water [3,4]. To overcome the limitations of gel treatment, researchers have developed preformed particle gels (PPGs), which hold significant importance in the area of oil field profile control and water shutoff.

**Figure 1:**
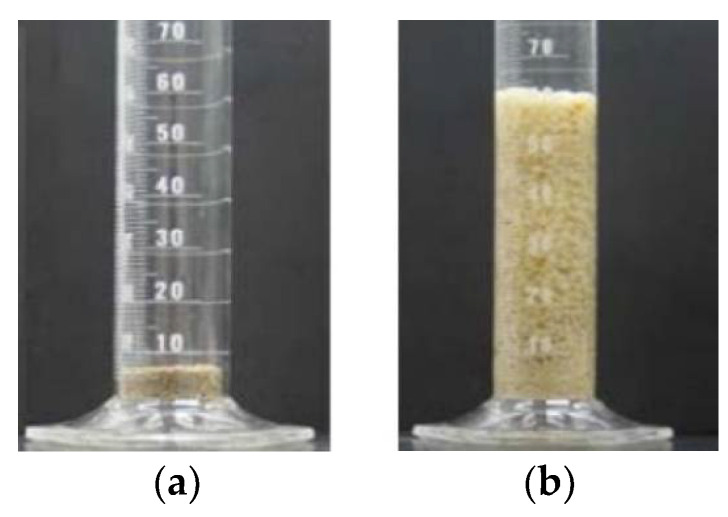
(**a**) Dried particles; (**b**) swelling particles [5].

PPGs possess strong hydrophilic groups (such as amide and carboxyl groups) and a three-dimensional network structure. When in contact with water, hydrogen bonds form between water molecules and the hydrophilic groups, causing the molecular chains between crosslinking points to unfold and generate cohesion. At equilibrium, PPGs can swell from several times to several hundred times their dry diameter. As shown in Figure 1. PPGs can be synthesized in several ways. 

The first method involves the formation of high-strength gel clusters through aqueous radical polymerization, followed by granulation, drying, crushing, and sieving to obtain solid particles. This process is simple and cost-effective. Another method involves obtaining PPG emulsions through inverse emulsion polymerization. The PPGs obtained using this method have small particle size, uniform distribution, and high roundness, but a higher cost [6]. The third method involves preparing bulk gels followed by shear cutting to obtain multiscale dispersed particle gels, characterized by low viscosity and negatively charged surfaces [7]. Since PPGs are prepared on the ground, their particle size and gel strength are controllable. 

During the process of PPGs absorbing water and swelling, when the water absorption reaches its maximum, the energy of the PPGs is at its lowest, indicating a stable state. The polymer chains constituting PPGs contain numerous detachable functional groups. After water absorption and swelling, they generate polymer anions and many positively charged cations. These cations disperse randomly around the anions, forming a stable electric field. When external cations are introduced, they shield the negative charges, reducing the intermolecular forces between polymer molecules, making stabilization easier and decreasing water absorption capacity. The influence of divalent cations on PPG performance is particularly significant. Compared to gels, they have better compatibility with formation water and shear resistance [8,9]. Unlike traditional polymer gels, PPGs exhibit a lower swelling ratio in high-salinity water, leading to an increase in crosslinking density and enhanced thermal stability. The introduction of inorganic fillers and molecular structure design can improve their temperature resistance by up to 120 °C [5,10,11,12]. The properties of PPGs are influenced by their composition, salinity, pH, and temperature. As the dosage of the main agent and crosslinking agent increases and the dosage of the initiator decreases, the swelling ratio of the PPGs decreases [13]. When the temperature increases, the swelling capacity of PPGs increases but their strength decreases. With an increase in the salinity of formation water, the swelling capacity of PPGs decreases while their strength increases. The influence of salinity on the plugging performance of PPGs is greater than that of particle size [14]. PPG suspensions contain only one effective component, simplifying injection equipment and reducing operational costs.

Mature oil fields with high water content commonly have high-permeability zones or fractures. PPGs of appropriate size and strength can preferentially enter high-permeability channels, causing minimal damage to low-permeability oil-producing layers. The main difference between the various types of PPGs lies in their particle size, ranging from nano- to millimeter-scale PPGs and microgels. Nanoscale PPGs and microgels can be used to plug pores with permeabilities less than 1 Darcy. Millimeter-scale PPGs can effectively block various fractures or high-permeability channels in reservoirs [5,15]. Key factors in selecting PPGs for application include compatibility with produced water, swelling ratio in injected water, strength after swelling and initial particle size. Additionally, combining with water flooding, gas displacement, and polymer displacement can enhance both the plugging performance of PPGs and the displacement efficiency of oil-displacing agents [16,17,18,19,20,21].

This paper first explains that PPGs, with their special properties, can be used for profile control and water shutoff in oilfields. It then introduces the mechanisms and application examples of PPGs for in selectively plugging high-permeability water flow channels in formations. The main focus of this study is to systematically summarize recent research into novel PPGs, aiming to identify the research directions of PPGs and the methods and strategies used for optimizing their performance, guiding their practical applications.

## 2. The Plugging Mechanism of PPGs

**Figure 2:**
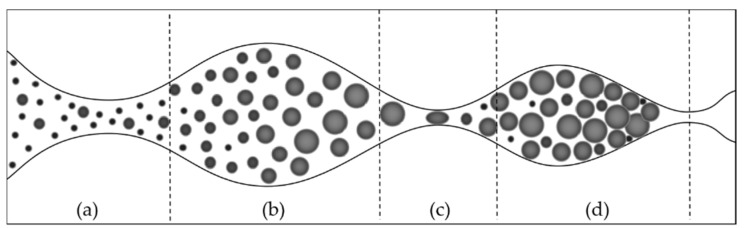
Migration of PPGs in formation channels [4]. (**a**) Passage of PPGs before swelling; (**b**) passage of PPG after swelling; (**c**) passage through pore throats after PPGs deformation or fragmentation; (**d**) lodging or entrapment of PPGs resulting in blockages.

After being injected into a formation, PPGs preferentially enter high-permeability channels under injection pressure, accompanied by swelling, fragmentation, and deformation. As the distance into the pores increases, the pressure on PPGs gradually decreases. When the resistance experienced by PPGs exceeds the driving force, they are retained, plugging the preferential water flow channels. As shown in Figure 2.

On a macroscopic scale, PPG migration in porous media has three modes: direct passage, passage after fragmentation, and blockage [22]. The greatest resistance that PPGs face during migration occurs at the pore throats. Expanded PPGs are elastic and can pass through pore throats smaller than their own particle size. During PPG migration, particle strength has a greater impact than particle size. Fully expanded PPGs have better injectability than partially expanded PPGs with a larger diameter [23,24,25]. In larger channels, PPGs can be retained by adsorption, and the smaller the PPG particle size, the higher the retention rate. When PPGs encounter pore throats, if the ratio of particle size to pore size becomes too large, interception occurs. Retention and interception lead to blockage, and the blockage rate under both modes decreases with increases in injection rate, formation permeability, and temperature [26].

Zhao et al. [27,28] indicated that the threshold penetration pressure (ΔPth) is a key factor influencing the selective injection of PPGs. ΔPth is the minimum driving pressure difference required for PPGs to enter channels or matrices, depending on the strength of the expanded PPGs and the ratio of particle diameter to pore size. For micrometer-scale PPGs in targeted formation channels, the ratio of particle diameter to pore size should be less than 2, while for non-targeted formations, the ratio should exceed 5 to prevent damage to the matrices. Dai et al. [29] revealed the matching pattern between the average diameter of PPGs and the size of pore throats by introducing a matching coefficient and using injection performance and regulation ability as indicators. In simulation core experiments conducted by Seright [30,31], tracer studies and permeability measurements indicated that PPGs are more efficient than gels in crack injection, are more conducive to plugging water in cracks, and cause less damage to low-permeability reservoirs. Coste et al. [32] discovered that after PPGs expand within channels, they can displace some of the residual oil within pores near the well zone. In distant well areas, where the pressure decreases, PPGs remain and to block the channels. Elsharafi et al. [33,34] discovered that high-strength PPGs cause less damage to low-permeability reservoirs compared to low-strength PPGs. Li et al. [35], using the Pressure Transfer Test (PTT), discovered that, when used to treat naturally fractured tight sandstone reservoirs, PPGs had a 12% higher plugging effect on vertical and oblique fractures compared to horizontal fractures. Moreover, the use of high injection pressure and low-salinity injection water could enhance the plugging effect.

## 3. Application Example

In 1999, Li et al. [36] demonstrated the feasibility of applying PPGs in the field of water shutoff and profile control. In the same year, the Zhongyuan Oilfield successfully used PPGs for the first time to adjust injection wells. The reservoir rock type was sandstone, with a reservoir temperature of 107 °C, total formation water salinity of 15 × 10^4^ mg/L, and an average permeability of 121 mD. The reservoir had been developed through water injection without hydraulic fracturing or the presence of natural fractures, and the injection well profiles showed strong reservoir heterogeneity. The candidate wells were two adjacent injection wells in Pucheng Oilfield: W51-75 and P-72. The average water cut of the corresponding production well prior to profile control was higher than 85%. After profile control with PPGs, the injection pressure of the injection wells increased, improving the injection profile. The daily oil production increased from 40 t/d to 60 t/d, with a total increased oil production of 3239 tons. Each ton of injected PPGs increased oil production by 158 tons, demonstrating favorable economic benefits. 

In 2000, Daqing Oilfield first utilized PPGs for profile control. The selected injection well was Xingbei Oilfield Well Xing-7-24. The reservoir temperature was 45 °C and the formation water salinity was 4500 mg/L. The corresponding four production wells had a water output exceeding 700 m^3^/d, with an average water cut exceeding 90%. Approximately 85% of the injected water directly exited through high-permeability zones that accounted for less than one-fifth of the reservoir thickness. This resulted in severely ineffective cycling. After trials, a total of 15.5 tons of 1.5–5 mm PPGs were injected into the well and the injection pressure increased from 5.0 MPa to 11.6 MPa. The water cut of the corresponding production well decreased by 8%, and the heterogeneity of the reservoir improved. The effectiveness lasted for over 6 months, with a total increase in oil production of approximately 2400 tons [37]. 

In a certain block of the Lamadian Reservoir in Daqing Oilfield, the reservoir temperature was approximately 40 °C, the formation water salinity was about 4000 mg/L, and the reservoir was highly heterogeneous. The average water cut in the production wells reached 95.4% and showed a rising trend. Between 2003 and 2004, WT (millimeter-scale PPGs) were used for conformance control in the four injection wells 7-1827, 7-1927, 8-1827, and 9-1927. These four water injection wells adopted the method of carrying PPGs with a polymer solution and cumulatively injected 132 tons of PPG suspension over a period of 5 months. After the PPG conformance control, the water injection profiles were improved. The oil production increased by 34.8 tons per day and the water cut decreased by 0.94%. By March 2005, the cumulative increase in oil production was about 15,000 tons, equivalent to 113 tons of oil per ton of PPGs used [38].

The Luo-1 Chang-8 block in Changqing Oilfield is a low-permeability reservoir which has been developed since 2007. Fractures are present in the central and northcentral areas of the block. By the end of 2011, the number of water-producing wells amounted to 38, accounting for 20.5% of the total wells, and indicating severe production capacity losses. In 2012, 17 injection wells in this block underwent conformance control. The injection process used “weak gel + PPGs + inorganic system” as the primary plugging components. After treatment, the average injection pressure of the 17 wells increased by 1.6 MPa, and 36 of the corresponding 93 production wells showed effective results. Cumulatively, these wells produced an additional 2733 tons of oil and showed a decrease of 1894 m^3^ in water production [39].

Alaska’s West Sak oil field had Void Space Conduits, which presented “short-circuiting” issues during water flooding. Traditional PPGs could plug pores, but had a short effective duration and lacked sufficient strength to control water flow. To enhance the plugging performance of PPGs, researchers developed a new type of re-crosslinkable preformed particle gel, RPPG [40]. Simulation core experiments showed that the breakthrough pressure in 2 mm fractures was more than five times that of conventional PPGs, with stability lasting up to 300 days. The target formation temperature in West Sak was approximately 24 °C, with permeability ranging from 20 to 3000 mD. Between 2017 and 2019, RPPG profile control treatments were conducted in the target formation. During the production process, no RPPGs were detected. The economic benefits of RPPGs were found to be 23% higher compared to traditional PPGs [41].

## 4. The Current Research Status

### 4.1. High-Temperature-Resistant PPGs

Traditional PPG, based on acrylamide homopolymer networks, dehydrates and degrades rapidly under high-temperature and high-salinity conditions [42]. There are typically three ways to enhance the thermal resistance of PPGs: copolymerizing with functional monomers, improving crosslinkers, and introducing inorganic materials. 2-Acrylamido-2-methylpropane sulfonic acid (AMPS) is a commonly used high-temperature-resistant and salt-resistant monomer; options also include N-vinyl pyrrolidone (NVP), sodium styrene sulfonate, 4-vinylimidazole, and vinylimidazole, which contain cyclic rigid groups. Using high-temperature-resistant crosslinkers can also enhance the thermal stability of PPGs.

Recent studies have found that chitosan can act as a crosslinker for high-temperature-resistant acrylamide-based gel [43,44], and Elaf et al. [45] developed biodegradable PPGs composed of polyacrylamide and chitosan cross-linkers, named PAM/Cs. Experimental verification confirmed that PAM/Cs exhibited excellent swelling performance and mechanical strength at specific compositional ratios and concentrations. However, their performance declined, and they were prone to degradation in high-temperature, high-salinity environments. Elaf et al. [46] subsequently incorporated N,N-methylenebisacrylamide (MBA) into the system and employed a microwave-assisted synthesis process to graft acrylamide (AM)/MBA onto the chitosan framework, thereby preparing environmentally friendly PPGs with temperature and salt resistance (Cs/PAMBAs). Its particle size range could be controlled between 10 μm and 1 mm. As a near-wellbore plugging agent, the swelling ratio in saline water with a salinity of 6 × 10^4^ mg/L was 11 within one hour. Cs/PAMBAs’ swelling performance and mechanical strength remained stable or slightly decreased under conditions of 200,000 mg/L salinity and temperatures ≤ 130 °C, indicating long-term stability.

**Figure 3:**
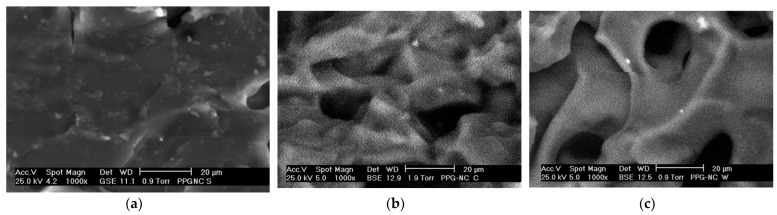
SEM image of modified PPG sample. (**a**) Dried sample; (**b**) sample swollen in production water; (**c**) sample swollen in distilled water [49].

The research conducted by Zhu et al. [47] indicated that the gel prepared from an AM/NVP/AMPS terpolymer using a high-temperature-resistant crosslinker exhibited excellent heat resistance. Ahdaya et al. [48] found that adding additives such as mica, walnut shells, and bentonite to PPGs significantly enhanced their strength. Durán-Valencia et al. [49] incorporated modified bentonite (MB) dispersion into the AM/NVP/AMPS system and synthesized modified PPGs through free radical polymerization at room temperature. This approach enhanced the mechanical strength of PPGs. SEM images before and after swelling are shown in Figure 3. In this system, the molar ratio of AM, NVP, and AMPS monomers was 1:1:1, with a 0.5% mass concentration of the crosslinker MBA, and a 2% concentration of modified bentonite (MB). When used at a concentration of 30%, it could remain stable for three months at 130 °C in brine with a salinity of approximately 25.5 × 10^4^ mg/L. Saghafi et al. [11] introduced N,N-dimethylacrylamide as a new monomer into the AM/NVP/AMPS system. The monomer ratio of AM/DMA/NVP/AMPS was set at 2:1:1:2, with a free radical polymerization mass concentration of 30%. Additionally, 0.45% MBA and 2.5% nano-clay was added to synthesize high-temperature-resistant PPGs. In formation water with a salinity of 22.5 × 10^4^ mg/L, the swelling ratio was 14, and the suspension maintained 75% of its original volume after aging for 120 days at 145 °C, indicating good temperature resistance. 

**Figure 4:**
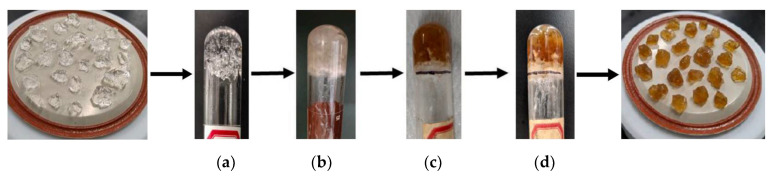
HT-PPG remains intact after aging at 150 °C for 18 months. (**a**) Before aging; (**b**) aging for 6 months; (**c**) aging for 12 months; (**d**) aging for 18 months [50].

Salunkhe et al. [50] used N,N-dimethylacrylamide and sodium styrenesulfonate (NaSS) as monomers, with divinylbenzene (DVB) as the crosslinker, and synthesized a high-temperature-resistant PPG (HT-PPG) after ratio optimization. Polydimethylacrylamide offered better thermal and hydrolytic stability than polyacrylamide, and DVB provided stable covalent crosslinks within the gel particles. After 18 months of aging at 150 °C, as shown in Figure 4, no degradation or dehydration was observeda nd the color change was due to residual oxygen. NMR spectra and SEM images indicated that the original chemical composition and crosslinking network structure were maintained even after aging; Schuman et al. [51] investigated the performance of HT-PPGs under North Sea reservoir conditions, where the formation water salinity was approximately 7.6 × 10^4^ mg/L, and the reservoir temperature ranged from 130 to 150 °C. The swelling ratio of HT-PPGs could reach over 30, and its stability lasted for more than 18 months, demonstrating excellent thermal stability and chemical stability. In simulation core experiments, HT-PPGs were able to reduce the permeability of 2 mm fractures to a millidarcy level.

### 4.2. Re-Crosslinkable PPG

**Figure 5:**
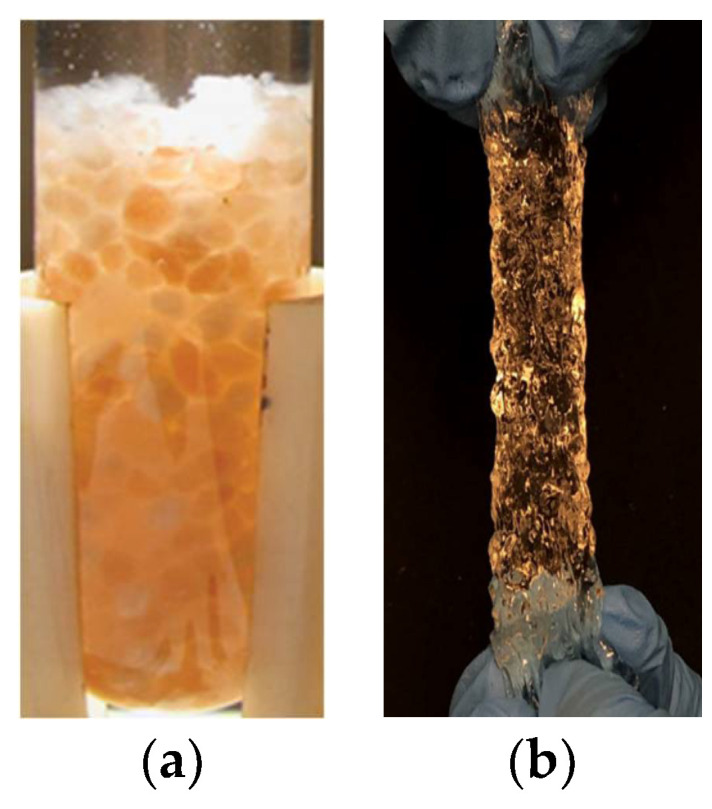
Comparison of morphology between PPGs (**a**) and RPPGs (**b**) [49,52].

Conventional PPGs, due to their lack of inter-particle cohesion and particle-rock adhesion, are prone to being flushed out by water flow in large fractures and high-permeability channels, thus limiting their plugging efficiency. RPPGs can undergo secondary crosslinking to form bulk gels, as shown in Figure 5, significantly increasing their plugging efficiency. Through molecular structure design, the addition of additives, or the use of multiple crosslinkers, PPGs can undergo secondary crosslinking after being injected into formations.

Branched PPG (B-PPG) are a type of PPG with branching structures between particles, which has excellent conformance control functionality in the “PPG–polymer–surfactant” ternary composite flooding system and has been successfully applied in the Shengli Oilfield [53,54,55,56]. Gong et al. [57] found in a sand pack plugging test that the B-PPG/polyacrylamide/surfactant system could further enhance oil recovery after polymer flooding. 

Xu Long et al. [58] introduced microbial polysaccharides (Diutan gum or xanthan gum) into B-PPG suspensions, enhancing the viscosity and stability of the system. A gel network based on double helix structures and gel particles could be formed between Diutan gum and B-PPG through van der Waals forces and hydrogen bonds. At low concentrations, Diutan gum could form a solid mesh layer on the surface of particles, enhancing the viscoelasticity, temperature resistance, and salt tolerance of the system. In heterogeneous sand pack heavy oil displacement experiments under conditions of 90 °C and a salinity of approximately 24,400 mg/L, the single B-PPG system was affected by high temperature and high salinity. The ultimate recovery ratio was 44.9%, only slightly higher than that of water flooding. However, the Diutan gum/B-PPG system showed a significant increase in injection pressure, leading to a final recovery ratio of 57.1%. Polysaccharide/B-PPG composite materials could form high-strength gels in high-permeability channels, demonstrating excellent conformance control performance.

Zhai et al. [59] added sodium d-gluconate (GA) and polyvinyl alcohol (PVA) to the acrylamide homopolymer system and developed temperature- and salt-resistant RPPGs (GA-RPPGs). The crosslinker system was composed of MBA, polyethylene glycol (400) diacrylate (PEG400DA), GA, and PVA. Under ultraviolet irradiation, MBA and PEG400DA formed a three-dimensional network structure of polymer chains, while the hydroxyl groups of GA molecules and the semi-interpenetrating structure of PVA also initiated crosslinking. At a certain temperature, GA molecules chelated with metal ions in saline solution, causing the boundaries of GA-RPPGs to disappear, and weak gel formation occurred through mutual stacking and contact. At 130 °C and in a 10% salt solution, GA-RPPGs exhibited good mechanical properties and re-crosslinking capabilities, retaining elasticity even after aging for 45 days.

Song et al. systematically evaluated two types of RPPGs: SR-RPPGs and HT-BRPPGs. When the concentration of divalent cations such as Ca^2+^ and Mg^2+^ in formation water was too high, the carboxyl groups in the hydrolyzed gel chelated with the divalent cations, causing severe synaeresis. The performance of conventional PPGs is also affected by excessive divalent cations. SR-RPPGs are suitable for plugging high-salinity fractured reservoirs [60]. SR-RPPGs were synthesized using AM and AMPS monomers, crosslinked with MBA. AMPS inhibited the hydrolysis of amide groups and enhanced thermal stability and salt tolerance [61]. During the production process, the addition of xanthan gum enhanced the system’s salt resistance and strength [62], while the addition of Cr^3+^ during injection facilitated re-crosslinking in fractures. Laboratory simulations of conditions in Middle Eastern fractured reservoirs (total salinity of formation water was approximately 21 × 10^4^ mg/L, with Ca^2+^ content of 19,000 mg/L and Mg^2+^ content of 2411 mg/L). SR-RPPGs with particle sizes of 1–2 mm were used, and the bulk gel formed after re-crosslinking could expand to over 30 times its original volume in formation water. After aging at 100 °C for 220 days, the strength grade was I, demonstrating good thermal stability and phase stability in Middle Eastern formation water and 5% CaCl_2_ solution. In cores with a fracture width of 1.5 mm, the breakthrough pressure gradient could reach 20.98 MPa/m, indicating excellent plugging performance.

HT-BRPPG were suitable for plugging high-temperature fractured reservoirs [52]. Laboratory simulations were conducted under the high-temperature sandstone reservoir conditions of the North Sea Ekofisk field, with a simulated formation water salinity of approximately 70,000 ppm. Within 2 h at 130 °C, HT-BRPPG exhibited a swelling ratio of up to 41, with minimal influence from formation water salinity and pH shown on its swelling performance. HT-BRPPG underwent transamidation reactions in the presence of branched polyethyleneimine for re-crosslinking, controlled by temperature. The bulk gel formed after re-crosslinking at 130 °C and aged for 450 days achieved a strength code of H. SEM images showed that it still retained a three-dimensional network structure, demonstrating excellent long-term thermal stability. In 3 mm fractured cement cores, the breakthrough pressure gradient was 8.77 MPa/m.

### 4.3. Delayed-Swelling PPGs

Conventional PPGs have a rapid water absorption rate, causing them to expand before entering formation pores, which affects the transportability of PPGs. This makes it difficult for PPGs to reach deep formations, reducing the efficiency of plugging. Using a dual crosslinker system of stable and degradable crosslinkers during PPG production could effectively delay the swelling time of PPGs. Additionally, incorporating nanoparticles also could enhance the delayed swelling property of PPGs [63].

Wu et al. [64] used degradable crosslinkers and hydrophobic monomers in the synthesis of PPGs, effectively delaying the swelling time of PPGs. Monomers included AM, AMPS, tert-butyl acrylate (tBA), and dimethylaminoethyl methacrylate (DMAEMA). The component ratio was AM:AMPS:DMAEMA:tBA = 4:1:1:1. MBA and polyethylene glycol (600) diacrylate (PEG600DA) were selected as crosslinkers, and salt-resistant delayed swelling PPGs were prepared through inverse emulsion polymerization. The inclusion of tBA monomers enhanced the hydrophobicity of PPGs, restricting their water absorption during transportation. Interaction between DMAEMA and AMPS increased the crosslinking network density and salt resistance. The use of dual crosslinker also increased the crosslinking network density of PPG. Upon entering the formation, the PEG600DA and tBA monomers hydrolyzed, restoring and enhancing the water absorption and swelling capacity of PPGs. At 90 °C, PPGs using only MBA as the crosslinker and PPGs using a dual crosslinker were dispersed in a sodium chloride solution with a salinity of 15 × 10^4^ mg/L. Two days later, the volume-swelling ratio of the dual-crosslinked PPGs remained at 2.36, while that of the single-crosslinked PPGs was close to 9. After twenty days, the volume-swelling ratio of the dual-crosslinked PPGs reached 9.49. The dual-crosslinked PPGs demonstrated excellent transportability and good plugging performance in formation pores.

In low-permeability reservoirs undergoing CO_2_ flooding, the presence of fractures can lead to gas channeling issues. Bai et al. [65] developed and evaluated a series of PPGs with controllable swelling rates, which could enhance the efficiency of CO_2_ flooding in reservoirs. Zhou et al. [66] utilized AM, sodium styrene sulfonate, and dimethyl diallyl ammonium chloride as monomers, while MBA and polyethylene glycol (200) diacrylate (UCA) were chosen as crosslinkers. Dual-crosslinked nano-particle microgels (DCNPM-As) were prepared by inverse microemulsion polymerization, exhibiting delayed swelling and anti-gas-channeling properties. The dual-crosslinking system could maintain smaller swelling ratios of DCNPM-As at low temperatures. UCA, as an unstable crosslinker, resulted in successive ester bond cleavage both externally and internally as the temperature increased (>80 °C). It exhibited secondary swelling characteristics, ultimately achieving higher swelling ratios compared to the microgels prepared using a single crosslinker. In an experiment involving supercritical CO_2_ flooding gas channeling resistance, DCNPM-As demonstrated a plugging efficiency of 95.48% and exhibited delayed swelling characteristics.

### 4.4. Augmented PPGs

Conventional PPGs are based on covalent bonds to form a crosslinked network structure, which may be susceptible to shear damage during transportation, affecting the plugging performance. Adding inorganic materials can improve the strength of PPGs (As shown in Table 1), but the enhancement is limited, and compatibility issues between the inorganic materials and polymers may arise during synthesis and use. Improving the crosslinking agent system of PPGs and designing their molecular structure can avoid compatibility issues while significantly enhancing their strength and toughness.

Ai et al. [76] synthesized a polyrotaxane crosslinker (PRc) and introduced a dynamic crosslinking structure into the acrylamide network, enhancing the deformability of acrylamide PPGs. In tensile experiments, the elongation at break of bulk gel prepared using MBA as the crosslinker was 332%, while that of bulk gel crosslinked by PRc (PR-G) reached 937%. After water absorption, the elongation at break of PR-G was 346%, demonstrating good tensile recovery. The crosslinking points of PR-G were on the cyclodextrin within the polyrotaxane crosslinker. When subjected to external forces, cyclodextrins could balance the tension on polymer chains like pulleys. At 90 °C, the swelling ratio in distilled water was 39.89, while in a 12% CaCl_2_ solution, it was 7.72. In a heterogeneous sand pack plugging test, PR-G exhibited good plugging capability for high-permeability layers.

Hao et al. [77] synthesized a host-guest inclusion supramolecular gel particle (S-PPG) using allyl-β-cyclodextrin (allyl-β-CD), cetyltrimethylammonium chloride (C16DMAAC), MBA, and AM as the main materials. The allyl-β-CD, as the host molecule, had an amphiphilic structure capable of encapsulating guest molecules such as C16DMAAC to form supramolecular gels. S-PPG possessed a synergistic crosslinking network structure of covalent and non-covalent bonds, which prevented it from breaking easily or self-bonding after fracture when passing through pores, ensuring its plugging performance. For the S-PPG system with a mass fraction of 0.6%, the ratios of host and guest monomers in the polymer were both 2.5%, with an average particle size of 34.56 μm. Under a strain amplitude of 1% and scanning frequencies ranging from 0.5 to 10.0 Hz, the elastic modulus was 73 Pa, which was 3.04 times that of conventional acrylamide PPGs. Its shear recovery performance and yield performance were four times that of conventional PPGs. After 24 h of swelling in formation water with a salinity of 6726 mg/L at 50 °C, the swelling ratio reached 23.

Deng et al. [78] developed a highly polymer-compatible crosslinker and enhancer: vinyl-functionalized silica nanoparticles (VSNPs). The polymer synthesis monomers included AM, N,N-dimethylacrylamide, and N-vinylpyrrolidone, with the addition of sodium alginate (SA). Using MBA and VSNPs as crosslinkers, a high-strength, high-temperature-resistant, and salt-resistant type of PPG (MC-PPG) was developed. MC-PPGs possessed a multi-crosslinking structure, including the nano-crosslinking of VSNPs, chemical crosslinking of MBA, self-crosslinking of DMA, and physical crosslinking of SA, which significantly improved their strength. DMA and NVP, as functional monomers, enhanced the temperature resistance and salt tolerance of MC-PPGs. MC-PPGs exhibited a honeycomb-like porous structure, with an equilibrium swelling ratio of up to 760% in deionized water. Compared to other PPGs, it had a higher elastic modulus (approximately 110 kPa) and stronger shear resistance and deformability. Additionally, the salinity and pH of the formation water had minimal effects on the swelling performance and strength of MC-PPGs. It remained stable for over 13 months at 120 °C and over 1 month at 160 °C. The high-temperature degradation products of MC-PPGs were liquid, causing minimal damage to the matrix. A core-flooding experiment demonstrated a breakthrough pressure gradient of 15.89 MPa/m at 20 °C and still exceeded 14 MPa/m at 120 °C, effectively plugging fractures and high-permeability channels.

### 4.5. Degradable PPG

During the oil recovery process, non-degradable substances can damage the reservoir [79].Conventional PPGs are difficult to degrade after plugging, and the residue can cause permanent damage to reservoirs. Degradable preformed particle gels (DPPGs) can degrade into low-viscosity liquids after completing plugging tasks, with minimal or no solid residues or by-products as temporary plugging agents in acidizing and fracturing operations.

Zhao et al. [80] introduced acid-resistant functional monomers and self-degradable crosslinking structures into PPGs, which could degrade into liquid after acidification. The DPPGs had an equilibrium swelling ratio of up to 70 in water and exhibited good plugging and self-degradation capabilities under acidic conditions in core experiments. The self-degradation time and strength could be controlled by adjusting the concentrations of monomers, initiators, and crosslinkers [81]. Zhang et al. [82] introduced sodium alginate (SA) into the polyacrylamide network, with MBA and Fe(NO_3_)_3_·9H_2_O as crosslinkers, to synthesize a DPPG (d-PPG). With the strain amplitude fixed at 0.5%, the elastic modulus increased proportionally within the scanning frequency range of 0.1–100 Hz, reaching up to 86,445 Pa. The equilibrium swelling ratio and swelling rate increased when the temperature rose and salt concentration decreased. Dried d-PPGs exhibited an equilibrium swelling ratio of approximately 375% after 5 h in a 50000 ppm NaCl solution at 80 °C, with a degradation time of 501 h. When the temperature rose to 100 °C, the degradation time decreased to 107 h. In core-flooding experiments, d-PPGs demonstrated a high breakthrough pressure, with a plugging efficiency of up to 99.83%. After degradation, the liquid viscosity was less than 5 mPa·s, causing minimal damage to the formation.

## 5. Discussion

PPGs’ mechanism of selective plugging of water channels differs from traditional polymer gels. Traditional polymer gel plugging agents form a gel in the formation pores, adsorbing onto the pore walls, and selectively plugging water relying on their property of “swelling in water and remaining unchanged in oil”. PPGs selectively enter high-permeability water flow channels, swell upon water absorption, and rely on adsorption, retention, and interception to achieve plugging. In the production of PPGs, the polymer crosslinking process occurs in surface equipment, with controllable particle size and strength, overcoming the challenges of precise control over gelation time, gel quality, and injection depth faced by traditional polymer gels. Moreover, PPG suspension has only one effective component, making injection equipment simple and production operation costs low.

The improvement of PPG performance can be achieved through several pathways. Firstly, the molecular structure design of polymers can enhance the strength and high-temperature resistance of PPGs. Molecular structure design involves copolymerization with monomers containing functional groups into the original polymer system. For example, the sulfonic acid group on the AMPS monomer provides large steric hindrance and electrostatic repulsion, enhancing the flexibility of polymer molecular chains and inhibiting polymer hydrolysis at high temperatures; cyclic rigid groups not only provide significant steric hindrance but also absorb heat from other parts of the molecule through resonance absorption.

Furthermore, PPGs can be endowed with special functionalities by improving the crosslinking agent system. The use of various specific crosslinking agents (such as the crosslinking agent system composed of MBA and Cr^3+^) can induce secondary crosslinking of RPPGs after injection into formation pores, forming bulk gels under environmental influence and greatly enhancing their plugging performance. The use of conventional crosslinking agents and degradable crosslinking agents can delay the expansion time of PPGs. The use of dual crosslinking agents allows PPGs to achieve a higher crosslinking network density before reaching the target formation. Upon reaching the target formation, one of the crosslinking agents undergoes degradation under environmental influence, enhancing the swelling capability of PPGs. Additionally, the use of multiple crosslinking agents or the introduction of special crosslinking structures can significantly enhance the strength and toughness of PPGs.

Lastly, adding a certain amount of inorganic material (such as nanoscale bentonite, etc.) can also enhance the thermal resistance and strength of PPGs. However, inappropriate amounts can lead to compatibility issues, adversely affecting the performance of PPGs.

In conclusion, the main approaches to improving the high-temperature resistance of PPGs are molecular structure design and improvements in the crosslinking agent system. It is worth noting that while functional monomers endow PPGs with excellent performance, the presence of large functional groups can have adverse effects on the polymerization process. Therefore, it is necessary to carefully select the optimal synthesis conditions when preparing PPGs. Re-crosslinkable PPGs, delayed swelling PPGs, augmented PPGs, and degradable PPGs are primarily prepared through the design of crosslinking agent systems. The addition of inorganic materials typically serves as auxiliary reinforcement. Finally, the performance evaluation of polymer gel and various PPGs is shown in Table 2.

## 6. Conclusions and Foresight

PPGs, relying on their controllable properties of swelling, size, and strength, can selectively enter and plug high-permeability zones in reservoirs. They are an important technology for profile control and displacement and water shutoff in oil fields. PPGs, with thier excellent performance and functionality, have been widely applied in oil fields with positive results.

Applicative prospects or research directions for PPGs:

(1) Exploring functional monomers and crosslinking agents resistant to high temperatures, targeting high-temperature reservoirs, and developing new types of high-temperature-resistant PPGs while ensuring plugging performance and long-term stability.

(2) PPGs are prone to being flushed out when dealing with large fractures, resulting in poor plugging effectiveness. High-strength RPPGs can effectively plug large fractures or excessively high-permeability channels.

(3) Conventional PPGs expand too rapidly in formation pores, lacking deep profile control. Delayed-swelling PPGs are easier to inject into deeper reservoirs, significantly enhancing plugging efficiency. 

(4) PPGs have potential applications in gas reservoir water shutoff.

## Figures and Tables

**Table 1:** Enhancing the performance of PPGs by introducing inorganic materials.

Researcher	Study on PPGs	Improvement
Kaio A. B. Pereira [67]	The Influence of Fly Ash (CFA) on Polyacrylamide Systems.	Viscoelasticity and temperature resistance are improved.
Peyman Abbasi Khoshkar [68]	The effect of nano-clay and nano-silica on AM-AMPS system.	The equilibrium swelling ratio and temperature resistance were improved, the pH and CO_2_ tolerance were improved, and the synaeresis of PPG was inhibited.
Abhinav Kumar [69]	The Reinforcing Effect of Nanomontmorillonite on Polyacrylamide Systems.	The swelling performance, thermal stability and strength are improved, and the water shutoff performance in sandstone is enhanced.
Aminsadegh Paprouschi [70]	The effect of Sodium silicate and graphene nanosheets on AM-AMPS system.	The storage modulus is greatly improved, and the strength, thermal stability, and dehydration tolerance are improved.
Sthéfany Z.S. do Amparo [71]	Graphene oxide- and carbon nanotube-reinforced polyacrylamide PPGs.	The shear deformation resistance and swelling performance are improved.
Li [72]	The effect of laponite on AM-AMPS system.	The swelling rate and mechanical properties are enhanced, and the thermal stability is also slightly improved.
Kang [73]	Effect of sawdust on acrylamide/acrylic acid system.	Viscoelasticity, shear resistance, and yield stress are improved, and the plugging rate in cracks is improved.
Abhinav Kumar [74]	Effect of halloysite nanotubes on polyacrylamide PPGs.	The swelling capacity, elastic modulus and temperature resistance are improved, and the plugging effect of permeability in the 2-6 D sand pack plugging test is enhanced.
Yugal Kishor Pandit [75]	Effect of bentonite and silica on AM-NVP-AMPS system.	Long-term thermal stability, elastic modulus and plugging performance are enhanced.

**Table 2:** Performance evaluation of polymer gels and various PPGs.

	Conventional Polymer Gels	Conventional PPGs	High-Temperature Resistant PPGs	Re-Crosslinkable PPGs	Delayed Swelling PPGs	Augmented PPGs	Degradable PPGs
Temperature tolerance	×	√	√√	√	√	√	√
Salt tolerance	×	√	√	√	√	√	√
Selective water plugging	√	√	√	√	√	√	√
Long-term stability	×	√	√	√	√	√√	×
Migration capability	×	√	√	√	√√	√	√
Convenient construction	×	√	√	√	√	√	√
Applicability of large fractures	×	×	×	√	×	×	×

‘×’ represents suboptimal; ‘√’ represents good; ‘√√’ represents excellent.

## Data Availability

No new data were created or analyzed in this study. Data sharing is not applicable to this article.
